# Antisense-Oligonucleotide Mediated Exon Skipping in Activin-Receptor-Like Kinase 2: Inhibiting the Receptor That Is Overactive in Fibrodysplasia Ossificans Progressiva

**DOI:** 10.1371/journal.pone.0069096

**Published:** 2013-07-04

**Authors:** SongTing Shi, Jie Cai, David J. J. de Gorter, Gonzalo Sanchez-Duffhues, Dwi U. Kemaladewi, Willem M. H. Hoogaars, Annemieke Aartsma-Rus, Peter A. C. ’t Hoen, Peter ten Dijke

**Affiliations:** 1 Department of Molecular Cell Biology, Cancer Genomics Centre Netherlands and Centre for Biomedical Genetics, Leiden University Medical Center, Leiden, The Netherlands; 2 Institute for Molecular Cell Biology, University of Münster, Münster, Germany; 3 Department of Human Genetics, Leiden University Medical Center, Leiden, The Netherlands; Children’s Hospital Los Angeles, United States of America

## Abstract

Fibrodysplasia ossificans progressiva (FOP) is a rare heritable disease characterized by progressive heterotopic ossification of connective tissues, for which there is presently no definite treatment. A recurrent activating mutation (c.617G→A; R206H) of activin receptor-like kinase 2 (ACVR1/ALK2), a BMP type I receptor, has been shown as the main cause of FOP. This mutation constitutively activates the BMP signaling pathway and initiates the formation of heterotopic bone. In this study, we have designed antisense oligonucleotides (AONs) to knockdown mouse ALK2 expression by means of exon skipping. The ALK2 AON could induce exon skipping in cells, which was accompanied by decreased ALK2 mRNA levels and impaired BMP signaling. In addition, the ALK2 AON potentiated muscle differentiation and repressed BMP6-induced osteoblast differentiation. Our results therefore provide a potential therapeutic approach for the treatment of FOP disease by reducing the excessive ALK2 activity in FOP patients.

## Introduction

BMPs are multifunctional growth factors that play key roles in bone formation, and heart and liver development [1], [2], [3]. The activity of the BMP pathway is precisely regulated to elicit its function in different cellular contexts. Perturbation of BMP pathways can lead to multiple diseases, including fibrodysplasia ossificans progressiva (FOP), a genetic disease caused by constitutively activated BMP signaling [4], [5], [6], [7].

FOP is a rare disease in which acute inflammation results in progressively ossified fibrous tissue. Minor traumas such as intramuscular immunization, muscle fatigue or muscle trauma from bumps or bruises can initiate the formation of heterotopic bones in the soft tissue [6]. Since surgical trauma also induces ectopic bone formation, surgery to remove ectopic bone is not an option for FOP patients. In the past decade, a variety of gene mutations in the activin receptor type IA/activin-like kinase 2 (ACVR1/ALK2) gene, encoding one of the type I BMP receptors, were found in most FOP patients [4]. The most common *ALK2* FOP mutation is a change of guanine (G) into adenine (A) causing an arginine to histidine substitution (R206H) in the ALK2 GS domain [4]. Due to this mutation, the FOP ALK2 shows a lower binding affinity for its negative regulator FKBP12, which results in elevated BMP signaling in cells, both in the presence and absence of exogenous BMP ligands [5], [8], [9].

The recurrent *ALK2* mutation in FOP patients provides a specific target for drug development. Plausible therapeutic approaches for inhibiting the excessive BMP signaling in FOP include ALK2 inhibitory RNA technology, anti-ALK2 monoclonal antibodies, and ALK2 small molecule inhibitors [10], [11]. Several small molecules already have been developed that efficiently inhibit ALK2 activity, such as dorsomorphin and LDN-193189 (LDN) [12], [13]. However, these compounds in addition also inhibit the activity of BMPR1 (ALK3), another type I BMP receptor [12], [13]. Other studies have suggested that dorsomorphin and LDN are not specific for BMP signaling as the inhibitors could block TGF-β-induced activity at higher concentrations [14]. The ideal BMP inhibitor for FOP patients would be an agent that normalizes the (excessive) ALK2 activity without affecting the functions of other kinases. Using the allele specific siRNA technique, two separate research groups have successfully obtained siRNAs that target the disease-causing ALK2, without affecting normal ALK2 expression [15], [16]. The siRNAs were used in cells from FOP patients to restore BMP activity and osteogenic differentiation [15], [16].

In addition to siRNAs, antisense oligonucleotides (AONs) mediated exon skipping might be a potential tool to modulate ALK2 activity. AONs are short synthetic, chemically modified single-stranded oligonucleotides between 20–30 base pairs in length. AONs can be used to modify splicing by specifically binding pre-mRNA sequences to block the access of spliceosome and other splicing factors, thereby excluding the target exon from the mature mRNA [17]. AON-mediated exon skipping has enabled the successful reframing of the mutated dystrophin mRNA and the restoration of dystrophin protein synthesis in skeletal muscle of Duchenne muscular dystrophy (DMD) patients [18], [19]. Systemic delivery of AONs is less challenging than for siRNAs, since AONs are single stranded, which is pharmacokinetically advantageous, allowing uptake by many tissues at significant levels after subcutaneous and intravenous administration without the need for specific formulations [20]. Therefore, adjustment of aberrant gene expression via exon skipping or RNaseH knockdown might be an attractive therapeutic option for genetic diseases.

In this study, ALK2 AON was designed to selectively modulate pre-mRNA splicing of mouse ALK2 to inhibit *Alk2* expression. The effects of ALK2 knockdown on ALK2-mediated BMP functions were assessed by analyzing myogenic differentiation and osteoblast differentiation. In line with the fact that BMP represses myogenic differentiation and potentiates osteoblast differentiation, we found ALK2 AON to potentiate myogenic differentiation of C2C12 myoblasts and inhibit osteoblast differentiation in mouse endothelial cells, suggesting that the endogenous BMP signaling in C2C12 cells and mouse endothelial cells were repressed by the ALK2 AON.

## Materials and Methods

### Antisense Oligonucleotides

ALK2 AON was specifically designed to target exon 8 of wild type mouse *Alk2*; AONs with full length phosphorothioate backbones and 2′-*O*-methyl-modified ribose molecules were obtained from Prosensa Therapeutics B.V. (Leiden, the Netherlands). AONs sequences are listed in Table 1.

**Table 1:** Antisense oligonucleotides used in this study.

AON	Sequences (5′-3′)	Targeting
ALK2 AON	GGGUUAUCUGGCGAGCCACCGUUCU	Mouse ALK2 exon 8
Control AON	UCAAGGAAGAUGGCAUUUCU	Control

### Cell Culture

Mouse C2C12 myoblast cells were maintained in DMEM medium (Gibco, Carlsbad, CA) supplemented with 10% fetal bovine serum (FBS) (Invitrogen, Carlsbad, CA), and penicillin/streptomycin (Invitrogen). For myogenic differentiation, C2C12 cells were cultured in DMEM (Gibco) supplemented with 2% FBS (Invitrogen). Mouse endothelial cells (MEECs [21] and 2H11 [22]) and mouse osteoprogenitor cells KS483 [23] were cultured in α-MEM (Gibco) and 10% fetal bovine serum (FBS) (Invitrogen), and penicillin/streptomycin (Invitrogen), the plates were pretreated with 0.1% gelatin (Sigma, St Louis, MO, USA). All of the cells were grown at 37°C in a humidified incubator with 5% CO_2_.

### AON Transfection

ExGen 500, the linear 22 kDa form of polyethyleneimine (PEI, MBI Fermentas, St.Leon-Rot, Germany), was used as a transfection reagent for KS483, MEECs and 2H11; DharmaFECT Duo (Thermo Scientific, Pittsburgh, PA, USA) was used as transfection reagent for C2C12 cells. Transfection was performed according to the manufacturer’s instructions.

### RNA Isolation and Quantitative Real-time PCR Analysis

Total RNA was isolated using the RNAII isolation kit (Machery Nagel, Düren, Germany) according to the manufacturer’s instructions. The RNA quantity and integrity were measured using RNA 6000 Nanochip in the Agilent 2100 bioanalyzer (Agilent Technologies, Amstelveen, the Netherlands). Reverse transcriptase-polymerase chain reaction was performed using the RevertAid H Minus First strand cDNA synthesis kit (Fermentas, St.Leon-Rot, Germany) according to manufactures instructions. Quantitative real-time PCR (qPCR) analysis was performed using the Roche LightCycler 480 and the relative expression levels of the genes of interest were determined in triplicate for each sample using the 2^−ΔΔCT^ method. Values were normalized to *Gapdh* expression, qPCR primers are listed in Table 2.

**Table 2:** Primers used in this study.

Primers	Sequences(5′-3′)	Used for
mouse ALK2 E7 FW	AAGTTGGCCTTATCATCC	ALK2 exon 8 skip PCR
mouse ALK2 E9 RV	GTACAATTCCGTCTCCCT	
mouse ALK2 FW	TGGCTCCGGTCTTCCTTT	ALK2 qPCR
mouse ALK2 RV	AGCGACATTTTCGCCTTG	
mouse GAPDH FW	AACTTTGGCATTGTGGAAGG	GAPDH qPCR
mouse GAPDH RV	ACACATTGGGGGTAGGAACA	
mouse BSP FW	AGGGAACTGACCAGTGTTGG	BSP qPCR
mouse BSP RV	ACACATTGGGGGTAGGAACA	
mouse OSC FW	AGACTCCGGCGCTACCT	OSC qPCR
mouse OSC RV	CTCGTCACAAGCAGGGTTAA	
mouse RunX2 FW	GAATGCTTCATTCGCCTCAC	Runx2 qPCR
mouse RunX2 RV	GTGACCTGCAGAGATTAACC	

### Immunofluorescence

Antibodies used for immunofluorescence were Desmin (Santa Cruz, Santa Cruz, CA, USA) and Myosin heavy chain (MF20; Developmental Studies hybridoma Bank, USA). The immunofluorescence procedure was performed as described previously [24].

### Alkaline Phosphatase (ALP) Assay

2×10^4^ MEECs cells were seeded in a 48-well plate. One day after AON transfection, cells were stimulated with TGF-β3 (kindly provided by Dr. K Iwata, OSI Pharmaceuticals, Melville, NY, USA) for 2 days. The ALP activity assay was performed after treatment with BMP6 in proliferation medium for another 2 days. KS483 cells were seeded in a 96-well plate with 5×10^3^ cells per well. Two days after AON transfection, cells were stimulated with 100 ng/ml BMP6 (R&D, Minneapolis, MN, USA) for further 2 days. Histochemical examination of ALP activity was performed using naphtol AS-MX phosphate (Sigma) and fast blue RR salt (Sigma), as described previously [5]. To quantify the data, the histochemically stained cell material was solubilized in 50 mM NaOH in ethanol, and absorbance was measured at 550 nm.

### Mineralization Assay

AON-transfected MEECs were stimulated with 5 ng/ml of TGF-β3 for 2 days in growth medium. Mineralization assay was performed after the cells were cultured in osteogenic medium, which is comprised of α-MEM supplemented with 5% FBS, 0.2 mM of ascorbic acid (Sigma), dexamethasone (Sigma) and 10 mM of β-glycerolphosphate (Sigma), containing 100 ng/ml of BMP6 for another 4 days. Confluent KS483 cells were transfected for 2 days, then stimulated with 100 ng/ml BMP6 for 4 days in growth medium. The mineralization assay was performed after subsequent 14 days of culturing in osteogenic medium, medium refreshed every 3–4 days. To visualize mineralization, cells were stained with 2% alizarin red S solution (Sigma).

### Transcription Reporter Assays

2H11 cells were seeded in 24-well plates, and then transfected with the BMP Responsive Element (BRE)-Luc reporter construct using PEI (Fermentas) as described previously [24]. After overnight serum starvation, the cells were stimulated with BMP6 (50ng/ml) for 8 hours. Harvested cells were assayed for luciferase activity with a Perkin Elmer luminometer. Each experiment was performed in triplicate and data represent the mean ± SD of three independent experiments with normalization to β-galactosidase activity.

### Western Blotting

Western blotting was performed as previously described using standard techniques [14]. The antibodies used for immunoblotting were phosphorylated Smad1/5/8 antibody (1∶1000, Cell signaling Technology, Danvers, MA, USA) and GAPDH antibody (1∶40,000, Sigma). GAPDH was used as the loading control.

## Results

### Design of AON for ALK2 Exon Skipping in Mouse Cells

**Figure 1:**
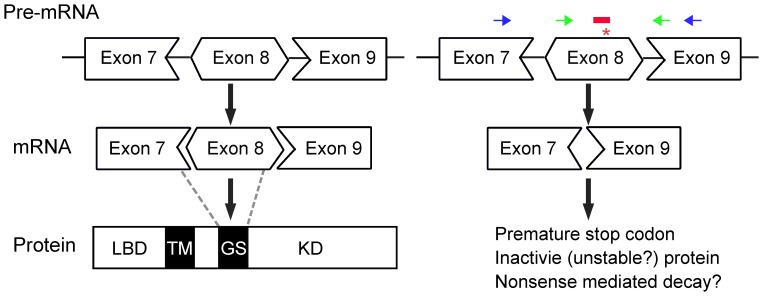
Schematic overview of ALK2 exon skipping. Left panel: The ALK2 protein structural domains include ligand binding domain (LBD), transmembrane domain (TM), GS domain (GS), and kinase domain (KD). The position of each exon relative to each domain is denoted by broken lines. Right panel: The FOP mutation hot spot (c.617G→A; R206H) is in exon 8 (*). ALK2 AON (red bars, the sequence covered the FOP mutation hot spot) hybridizes and hides exon 8 from the splicing machinery, resulting in skipping exon 8 upon mRNA splicing. This out-of-frame mutation will lead to non-sense mediated decay of *Alk2* mRNA. qPCR primers to detect Alk2 expression are indicated by green arrows; The primers for detecting of skipped band are indicated by blue arrows. While we targeted exon 8 for exon skipping, we do not exclude that other AONs targeting other exons can be designed that also inhibit ALK2 expression.

The classic FOP mutation, a G-A substitution in exon 7 of the human *ALK2* gene, leads to a R206H substitution in the GS domain of ALK2 protein, causing elevated BMP signaling in FOP patients [4], [5]. The mutated ALK2 exon sequence is highly conserved between mouse and human, and the exon 7 in human corresponds to exon 8 in mouse [25]. In an attempt to modulate ALK2-mediated BMP signaling, an ALK2 AON was designed to specifically target exon 8 encoding the GS domain of mouse ALK2 and the sequence of ALK2 AON overlapped with the location of hot spot mutation in FOP (Figure 1). Upon transfection and entry into the cell nucleus, the AON was anticipated to modulate mouse ALK2 pre-mRNA splicing by masking and subsequent skipping of exon 8, which disrupts the reading frame (exon 8 is 100 base pairs, not dividable by three) (Figure 1). We have chosen AON against exon internal site as we demonstrated previously that they outperform AONs targeting splice sites [26]. The truncated ALK2 mRNA without exon 8 may be degraded via nonsense-mediated decay due to the introduction of a premature stop codon. The locations of primers to detect the skipped exon are indicated as black arrows in Figure 1.

**Figure 2:**
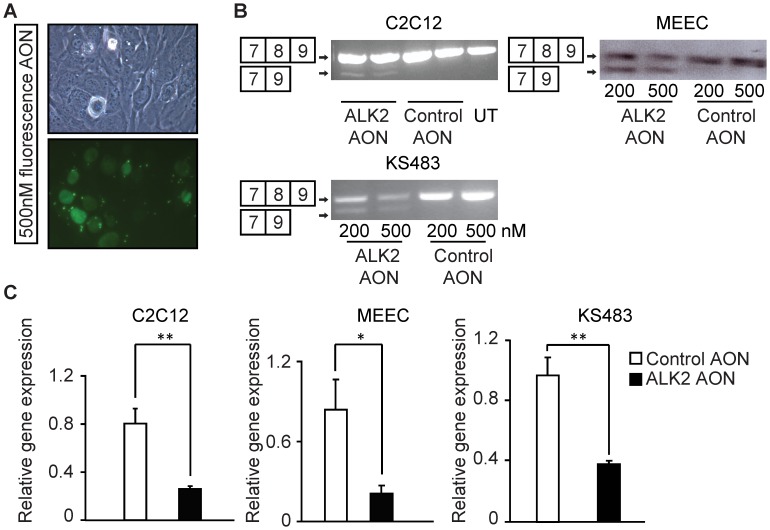
AON-induced ALK2 exon 8 skipping in various cell types. (A) C2C12 cells were transfected with 500 nM non-targeting, fluorescently-labeled AONs fluorescent AON in differentiation medium and fluorescent images were taken 2 days after transfection. (B) Various of cells were transfected with indicated control AON or ALK2 AON. RNA was isolated 2 days after transfection, and RT-PCR was performed to visualize the full length Alk2 (composed of exon 7, 8 and 9); and skipped Alk2 (composed of exon 7 and exon 9). (C) MEECs cells were transfected with 100 nM control AON or 100 nM ALK2 AON, RNA was isolated 1 day after transfection. C2C12 cells and KS483 cells were transfected with AONs in proliferation medium; RNA was isolated 2 days post transfection. cDNA was synthesized using random hexamer primers and used for the quantification of *Alk2* expression. The relative full length ALK2 mRNA expression was analyzed. Alk2 RNA level was normalized to *Gapdh*; the level of expression for untreated sample was defined as 1. Values and error bars represent the means ± SD of triplicates. Statistical analysis was performed using Student’s t-test. *P<0.05, **P<0.005.

To test whether the designed ALK2 AON could induce exon skipping and decrease full-length ALK2 expression and/or ALK2 activity in cultured cells we applied ALK2 AON in various cell types. These cells were shown to be transfected with AONs with high efficiency (>70%) as visualized by the 5′-fluorescein (FAM)-labeled control AON (Figure 2A). RT-PCR on RNA harvested 2 days after transfection showed a skipped band representing the transcript without exon 8 upon transfection of the ALK2 and not the control AON (Figure 2B). qPCR analysis showed that *Alk2* expression was decreased about 70–80% in the cells treated with ALK2 AON (Figure 2C).

### Exon-skipping in ALK2 Reduced *Alk2* Expression and Potentiated Muscle Differentiation

**Figure 3:**
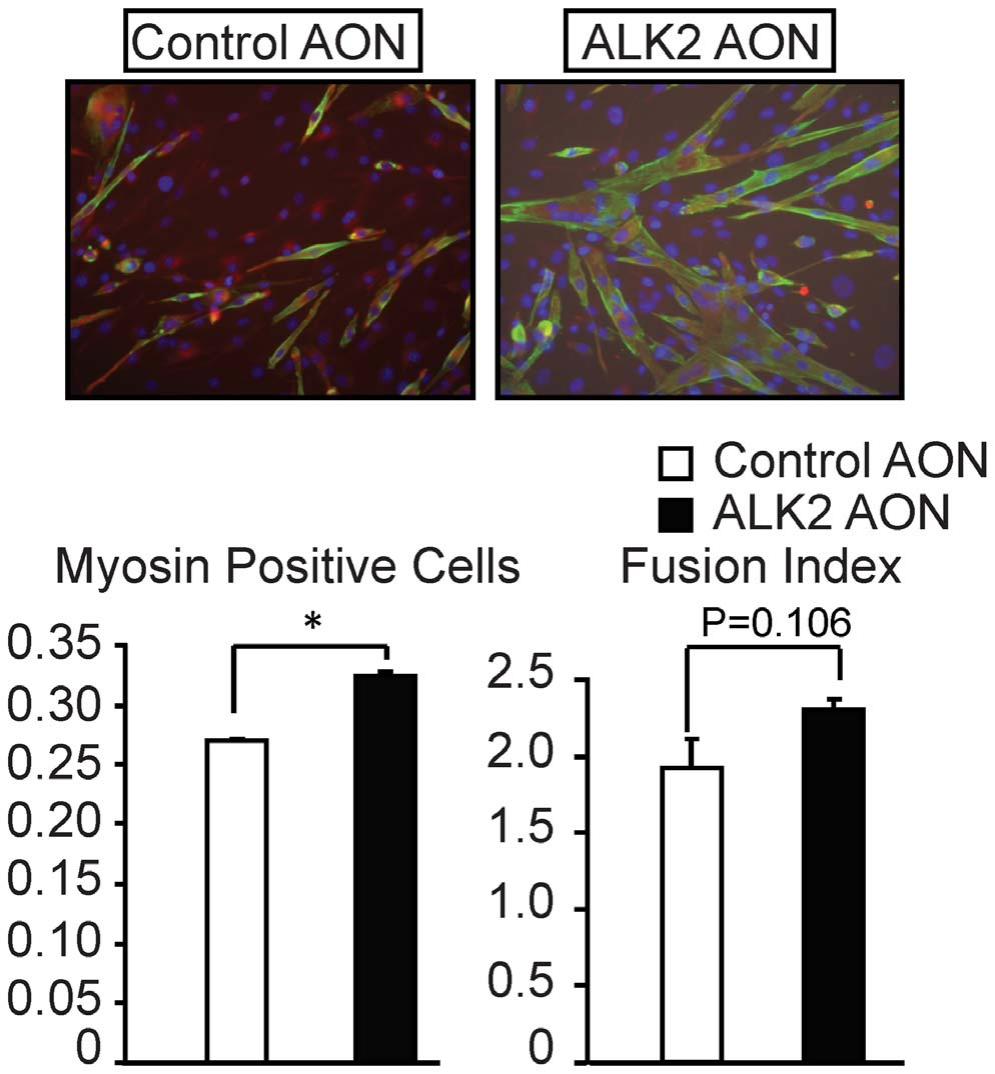
ALK2 AON enhanced myogenic differentiation in C2C12 cells. C2C12 cells were transfected with 200 nM control AON or 200 nM ALK2 AON in differentiation medium for 7 days. Then cells were immunostained with myosin and desmin. Myosin (green) was used to label the differentiated cells while desmin (red) was used to label myogenic cells. The values for the percentage of myosin positive cells or for the fusion index represent the average levels of two independent samples. Values and error bars represent the means ± SD. Statistical analysis was performed using Student’s t-test, using the untransfected samples as reference. *P<0.05.

BMP signaling is known to repress myogenic differentiation, and BMP inhibitors have been shown to potentiate the differentiation of myoblasts into myotubes [24], [27]. We therefore examined whether the ALK2 AON can decrease BMP signaling and function as a BMP inhibitor to potentiate myogenic differentiation of C2C12 myoblasts. Seven days after transfection, cells were fixed and immunostained for myosin to visualize the differentiated myotubes. The degree of differentiation was measured by determining the differentiation and the fusion indexes. The differentiation index was calculated as the percentage of myosin-positive cells out of all myogenic (desmin-positive) cells. The fusion index was calculated as the average number of nuclei in the differentiated myotubes. The ALK2 AON was found to enhance both the differentiation index and the fusion index in C2C12 cells (Figure 3), suggesting that AON-mediated ALK2 knockdown can potentiate myoblast differentiation via repression of endogenous BMP signaling.

### ALK2 AON-mediated Exon Skipping Decreased BMP Signaling and Mineralization in Endothelial Cells

**Figure 4:**
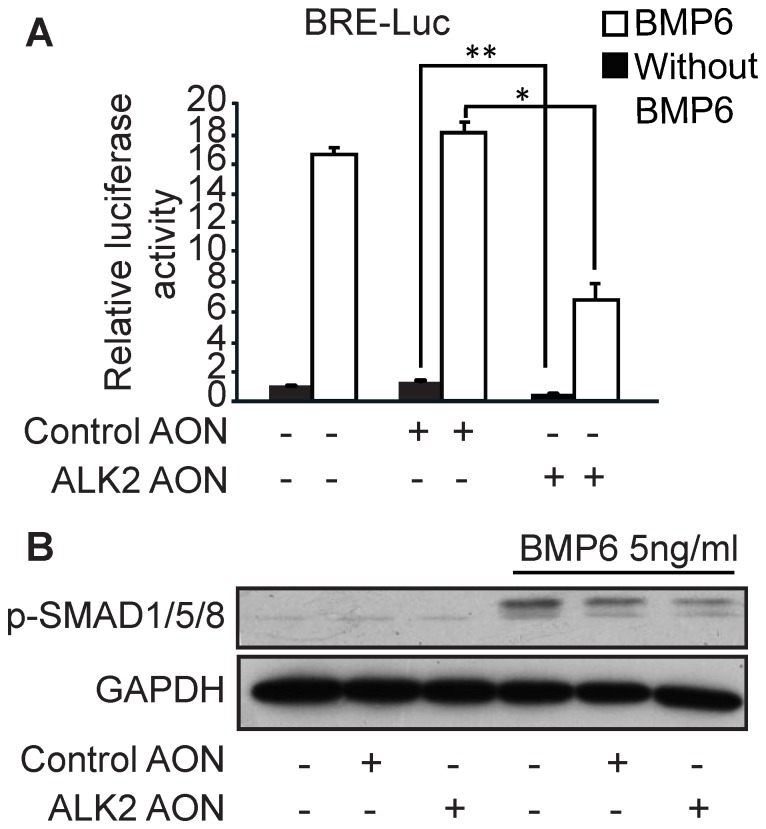
ALK2 AON-induced exon skipping decreased BMP signaling in 2H11 endothelial cells. (A) 2H11 cells were cotransfected with 100 nM of the indicated AON and the BMP reporter construct BRE-Luc for 16 hours. Subsequently, cells were starved for 8 hours, and then stimulated with 50 ng/ml BMP6 overnight. Luciferase reporter activity was measured after stimulation and normalized with β-gal activity. (B) 2H11 cells were transfected with 100 nM control AON or 100 nM ALK2 AON in proliferation medium. One day after transfection, the cells were serum starved for overnight and stimulated with 5 ng/ml BMP6 for 1 hour. Protein was isolated and western blotting was performed to check the Smad1/5/8 phosphorylation. GAPDH was used as loading control. Data are means ± SD from three independent experiments. Statistical analysis was performed using Student’s t-test, using the untransfected samples as reference. *P<0.05, **P<0.005.

Recently, endothelial cells were reported as potential osteoprecursor cells for ectopic bone formation in FOP patients [28], [29]. Therefore, our next step was to test whether the ALK2 AON can repress BMP signaling and osteoblast differentiation in endothelial cells. The ALK2 AON (and not a control AON) decreased BMP-induced BMP-responsive element (BRE)-driven luciferase reporter activity (Figure 4A). BMP6-induced Smad1/5 phosphorylation was also inhibited by the ALK2 AON, albeit weakly (Figure 4B).

**Figure 5:**
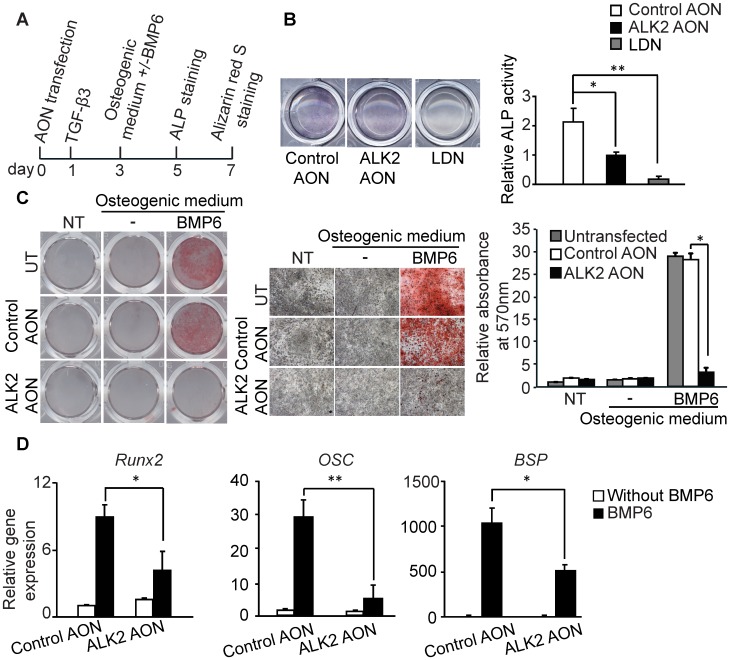
BMP-induced osteoblast differentiation was impaired by ALK2 AON-induced exon skipping in MEECs. (A)As a mineralization assay, MEECs were seeded into a 24-wells or 48-wells plate. One day after transfection, cells were stimulated with 5 ng/ml TGF-β3 for 2 days and switched to osteogenic medium with or without 100 ng/ml BMP6 for several days (with medium refreshment after 4 days). ALP staining was performed 2 days after maintaining in osteogenic medium. After 4 days in osteogenic medium, the cells were fixed and stained with 2% alizarin red S solution for mineralization staining. (B) MEECs were transfected with 200 nM control AON or 200 nM ALK2 AON, and stimulated with osteogenic medium indicated in figure 4A. The LDN sample indicates LDN-193189 was present during the whole experiment. Representative microscopic pictures of staining (upper panel) are shown; ALP activity was represented as the average of three independent samples, and was normalized by protein concentration. (C) MEECs were transfected with 100 nM control AON or 100 nM ALK2 AON in grow medium and treated as in panel 4A. The mineralization was visualized by alizarin red S staining. The plate was scanned (4C, left panel), and scanned under a microscope (4x, 4C, middle panel). Alizarin red S staining was quantified by stain extraction and absorbance reading at 570 nm (4C, right panel). (D) RNA was collected in experiment 4C. mRNA expression of *Runx2*, *OSC* and *BSP* was measured by qPCR. All the experiments were performed for three times and were normalized to *Gapdh*. Each value is the means ± SD. Statistical analysis was performed using Student’s t-test, using the untransfected samples as reference. *P<0.05, **P<0.005.

We next evaluated the therapeutic potential of the ALK2 AON in the treatment of excessive bone formation such as occurring in FOP patients. For this purpose we investigated the effect of the ALK2 AON on endothelial to osteoblast transdifferentiation by using MEECs cultured under osteogenic conditions. MEECs were chose due to the short transdifferentiation period. Transfected MEECs were treated with TGF-β3 for 2 days and then refreshed with osteogenic medium with or without BMP6 for several days (Figure 5A). The osteoblast differentiation can be measured by determining alkaline phosphatase (ALP) activity, an early marker for osteoblast differentiation. Histochemical staining revealed that ALP activity in ALK2 AON treated cells was significantly decreased (Figure 5B). Compared to LDN-193189 treated sample in which most of the ALP activity was blocked, ALP activity in ALK2 AON treated cells was only partly blocked (Figure 5B). Furthermore, we analyzed the effect of ALK2 AON on osteoblast differentiation by alizarin red S staining, a staining to detect calcium mineralization. BMP6 induced mineralization was significantly decreased by exon skipping of ALK2 (Figure 5C). Consistent with the ALP activity and alizarin red S staining results, qPCR analysis confirmed that exon skipping in ALK2 can decrease the expression of *Runx2,* bone sialoprotein (*BSP*) and osteocalcin (*OSC*) (Figure 5D). Together these data indicate that ALK2 AON can decrease BMP-induced osteoblast differentiation.

### The ALK2 AON Decreased BMP6-induced Osteoblast Differentiation in KS483 Osteoprogenitor Cells

**Figure 6:**
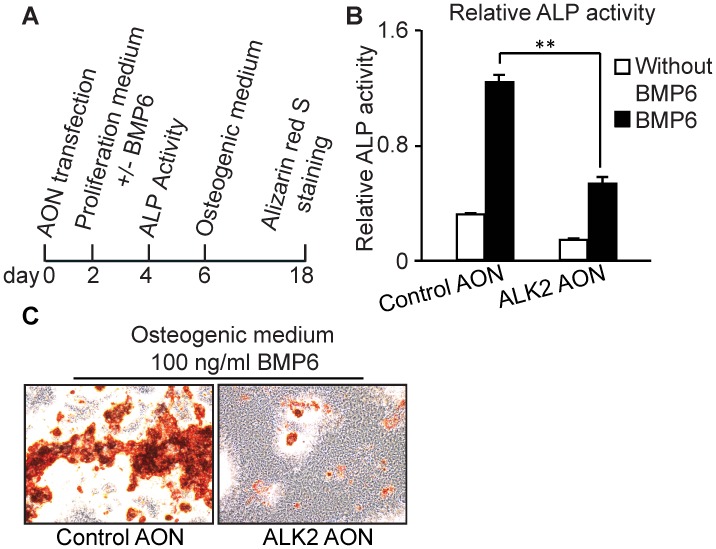
ALK2 AON reduced BMP-induced osteogenic differentiation in KS483. (A) Confluent KS483 cells were transfected for 2 days in a 96-wells or 24-wells plate. Two days after AON transfection, cells were stimulated with 100 ng/ml BMP6 (R&D, MN, USA) for 2 additional days and ALP assay was performed. For mineralization assay, after stimulation with 100 ng/ml BMP6 (R&D, MN, USA) for 4 days, cells then switched to osteogenic medium for subsequent 14 days. Medium was refreshed every 3–4 days. (B) Confluent KS483 cells were transfected with 200 nM control AON, or 200 nM ALK2 AON for 2 days in proliferation medium. Then cells were stimulated with 100 ng/ml BMP6 for another 2 days in proliferation medium. Cells lysates were harvested and ALP activity was measured. Data are presented as means ±SD. (C) Confluent KS483 cells were transfected with 200 nM control AON, or 200 nM mouse ALK2 AON for 2 days in proliferation medium. The cells were then stimulated with 100 ng/ml BMP6 in proliferation medium for 4 days. Then cells were maintained in osteogenic medium for another 12 days. Medium was refreshed every 3–4 days. The cells were finally stained with alizarin red S solution to visualize the mineralized area in KS483 cells. Statistical analysis was performed using Student’s t-test, using the untransfected samples as reference. **P<0.005.

In addition to endothelial cells, mesenchymal stem cells are considered as another source of osteoprecursors responsible for BMP induced ectopic bone formation in mice [6], [30]. In FOP patients, the mesenchymal stem cell-like cells derived from endothelial cells are considered to be in part responsible for heterotopic ossification [29]. Pluripotent mesenchymal stem cells KS483 cells can be differentiated into osteoblasts, chondrocytes and adipocytes *in vitro* [31], [32]. Two days after transfection, KS483 cells were maintained in proliferation medium with or without BMP6 for 2 days before measuring ALP activity. For alizarin red S staining, transfected cells were cultured in proliferation medium for 4 days and then refreshed with osteogenic medium with or without BMP6 for 12 days (Figure 6A). The ALK2 AON also efficiently repressed BMP6-induced osteoblast differentiation in KS483 cells, as visualized by the ALP activity (Figure 6B) and the mineralization assay (Figure 6C). qPCR analysis confirmed that exon skipping in ALK2 can decrease the expression of BMP6-induced osteogenic gene expression (data not shown).

## Discussion

In this study, an ALK2 exon-skipping AON was designed based on previous published guidelines [33]. The mouse ALK2 AON we designed can specifically induce skipping of exon 8 in different cell types, including C2C12 myoblasts, KS483 osteoprogenitor cells, and two types of endothelial cells (2H11 and MEECs). The removal of exon 8 might cause the transcripts to be degraded via nonsense-mediated decay. We observed a weak skip product in the RT-PCR analysis, which suggested that the transcript with premature stop codon might not be stable. Potentially, the truncated protein, if stably produced, could have dominant negative receptor activity. However, high amounts of kinase inactive ALK2 are required to achieve dominant negative effects in cultured cells, while the accumulated mutant ALK2 in AON transfected cells may be readily degraded. From studies examining the effect of mismatches on efficiency, we know that AONs with 1 mismatch at the 5′ of 3′ end of the AON can still work, but these AONs may show a reduced efficiency. More mismatches leads to poor or inefficient AONs [34]. As there is no complete overlap of ALK2 AON with another region in the genome as determined by blasting the ALK2 AON sequence with the full genome, and ALK2 AON did not inhibit the expression of other related type I receptors, such as ALK3 and ALK1 (or housing keeping genes) (data not shown), we conferred that ALK2 AON specifically targets ALK2 *in vitro*.

Importantly, the ALK2 AON also can downregulate ALK2 and BMP-induced osteoblast differentiation in endothelial cells, which has recently been reported to be the major bone progenitor cell population in FOP patients [29]. Endothelial cells were first found to dedifferentiate into a mesenchymal stem cell-like phenotype by endothelial-to-mesenchymal transition, and subsequently to differentiate into cartilage and bone [29]. The ALK2 AON downregulated the levels of ALK2 mRNA and significantly reduced BMP-induced signaling responses and osteogenic differentiation in MEECs and more mature 2H11 cells. The effect of the ALK2 AON on BMP-induced Smad1/5 phosphorylation was relatively weak compared to other responses. This could be because of the different thresholds required for BMP-induced responses; BMP6-induced Smad1/5 phosphorylation as measured in a total cell lysate at the time point examined may need more efficient knockdown to observe a strong effect.

BMPs transmit signals through induction of heterotetrameric complexes consisting with type I receptors and type II receptors [7]. Utilization of type I receptors differs depending on BMP ligands; BMP-6 binds principally to ALK2, but also ALK3, ALK6 [35]. The role of ALK2 played in BMP6 induced osteoblast differentiation may be different depending on the cell types [9], [36]. While LDN can strongly block the BMP signal pathway and osteoblast differentiation (Figure 5B), ALK2 AON has a slightly lower inhibitory effect, which may explained by the minor role ALK2 played in BMP signal pathway in endothelial cells. Alternatively, as AON-mediated depletion of ALK2 does not affect the expression ALK3, the cells with ALK2 knockdown may still partly respond to BMP6 by signaling via ALK3 or other BMP type I receptors.

Our research provides a new approach for the therapy of FOP. The next step is to test whether the ALK2 AON can efficiently decrease ALK2 expression and ALK2-mediated BMP signaling *in vivo*. In this respect, it will be of great interest to test whether the ALK2 AON can inhibit the heterotopic ossification in ALK2 R206H knock-in mice that have recently been developed [37]. The specific chemistry of the type of AONs used in this study (2′-*O*-methyl phosphorothioate RNA) enhances nuclease resistance and stability of the AON-target RNA duplex [38]. The phosphorothioate backbones of AONs also prevent renal clearance [38], resulting in a long serum half-life and uptake by most of tissues. Phase 3 clinical trials are currently ongoing for DMD [39]. The research of applying AON to DMD holds a promise for the application of AON in FOP. The final goal would be to examine whether ALK2 AON can counteract the excessive BMP activity in tissues from FOP patients. We have attempted to specifically target the ALK2 R206H allele with AON-mediated strategies by human ALK2 AON, but so far this has not been successful. Taken together, the results presented here underscore that AONs have potential for the treatment of FOP, although more studies should be performed in this sense.
